# Emergency department waiting room: many requests, many insured and many primary care physician referrals

**DOI:** 10.1186/1865-1380-6-35

**Published:** 2013-10-01

**Authors:** Michael F Kamali, Minal Jain, Anunaya R Jain, Sandra M Schneider

**Affiliations:** 1 Department of Emergency Medicine, University of Rochester Medical Center, 601 Elmwood Ave, Box 655 A, Rochester, NY 14642, USA; 2 Department of Neurosurgery, University of Rochester Medical Center, 601 Elmwood Ave, Box 670, Rochester, NY 14642, USA

**Keywords:** Emergency department, Survey, Patients, Primary care, Satisfaction, Preferences

## Abstract

**Background:**

Increase in waiting time often results in patients leaving the emergency department (ED) without being seen, ultimately decreasing patient satisfaction. We surveyed low-acuity patients in the ED waiting room to understand their preferences and expectations.

**Methods:**

An IRB approved, 42-item survey was administered to 400 adult patients waiting in the ED waiting room for >15 min from April to August 2010. Demographics, visit reasons, triage and waiting room facility preferences were collected.

**Results:**

The mean age of patients was 38.9 years (SD = 14.8), and 52.5% were females. About 53.8% of patients were employed, 79.4% had access to a primary care physician (PCP), and 17% did not have any medical insurance. The most common complaint was pain. A total of 44.4% respondents reported that they believed their problems were urgent and required immediate attention, prompting them to come to the ED, while 14.6% reported that they could not get a timely PCP appointment, and 42.9% were actually referred by their PCP to come to the ED. About 57.7% of patients considered leaving the ED if the waiting times were too long. The mean acceptable waiting time before leaving ED was 221 min (SD = 194; median 180 min, IQR 120–270). A total of 39.1% survey respondents reported being most comfortable being triaged by a physician. Respondents were least comfortable being triaged by residents. On analyzing waiting room expectations for the survey respondents, we found that 70% of the subjects wanted a better estimate of waiting time and 43.5% wanted better information on reasons for the long wait.

**Conclusion:**

Contrary to popular belief, at our ED a large proportion of low-acuity patients has a PCP and is medically insured. Providing patients with appropriate reasons for the wait, an accurate estimate of waiting time and creating separate areas to examine minor illness/injuries would increase patient satisfaction within our population subset.

## Background

The wait to see a physician in the Emergency Department (ED) has been well documented in the literature since the early 1970s [1,2]. In today’s evolving scenario of health care, EDs are not only a point of care for the acutely ill, but they also serve the role of a safety net to provide health care to people regardless of their insurance status or ability to pay [3-5]. Other than the perceived acuity of illness, factors such as the convenience offered by the ED over PCP offices, lack of timely appointments with PCPs and inability to determine the severity of disease prompt even low-acuity patients to visit the ED [6-9]. This has contributed to a significant increase in the number of patients presenting to the ED, with nearly 123 million ED visits in 2008 in the US—an increase of over 23% over the past decade.

While patients with life-threatening illnesses are seen promptly, overcrowded EDs mean those with less acute illnesses wait for increasingly longer times. Lengthy wait times decrease patient satisfaction and cause some to leave without being seen [10]. The median waiting time in the ED was 35 min, and nearly 9% of the patients left without being seen by a physician in 2008 [11]. Many of these patients are less likely to return to that ED, which translates into an economic loss for the hospital and physician group [12].

Patient satisfaction in the ED has been associated with not only the waiting time, but also the quality of care provided by physicians, nursing and ancillary healthcare staff [13]. A study reported that patients in US EDs assigned 59% of their overall satisfaction to physician and nursing service and the remaining to waiting time [13]. Surveys done in other developed countries have also revealed that a majority of patients waiting in the ED consider competence or explanation by medical staff as one of the most important features that they value during their ED visit [14].

Our ED sees a cross section of socioeconomically and racially diverse populations. We wanted to understand the reasons why low-acuity adult patients visit our ED and identify their preferences and expectations while they are waiting to be seen.

## Methods

This survey was administered to a convenience sample of 400 patients in the ED waiting room between 12 p.m. and 12 a.m. from 15 April 2010 to 2 August 2010. Our 120-bed academic tertiary care ED has an annual volume of nearly 95,000 patients. The ED is organizationally divided into different wings for minor injuries/minor illnesses, patients waiting for inpatient beds, trauma/critical care, adult ED patients and pediatric ED patients. There is a separate observation unit and triage areas that are managed by the ED to care for patients. The ED also has separate entrances and registration desks for self-arrivals and patients brought in by EMS. Median time for all ED patients from arrival to provider irrespective of severity over the duration of the survey was 41.4 min. We defined lower acuity patients as those that were waiting more than 15 min from the time of triage. Two trained research assistants administered the survey in the ambulatory waiting area. Patients arriving by EMS who were brought to the ambulatory waiting area because of lower acuity of illness were also eligible for the survey.

A 42-item questionnaire was created in English language, with questions pertaining to demographic details (age, gender, race and ethnicity), employment, insurance status, reasons for current ED visit, ED experiences, triage preferences and waiting room facility preferences. All of the questions were close-ended except for the question on reasons for the ED visit. The Likert scale (0–5; 0 being very uncomfortable, 5 being very comfortable) was used to assess patient preferences. The survey was pilot tested twice among graduate students and EM residents, and appropriate changes were made before finally distributing the survey to the patients. The changes included re-formatting some questions for clarity so that people with a basic level of health literacy could understand it.

The study was approved by the Institutional Review Board. Two research personnel identified the waiting time for patients through the electronic medical record system. Patients above 17 years of age who had been waiting for 15 min or longer post-registration/triage to see a physician were approached for participating in the survey. All patients who had previously participated in the survey at an earlier time were excluded. A letter of information outlining the study protocol and potential risks was provided to these eligible patients. After obtaining verbal consent, the research patients were asked to fill out the paper printed questionnaire and submit it in a closed drop box within the waiting room. No identifying information was obtained. Patients were allowed to leave answers to questions blank. In case the patients did not understand a survey question, the research assistant provided explanations at the patient’s request only. If a research subject was called to the treatment area while filling out the survey, they were allowed to complete it inside the ED as well.

Statistical analysis was performed using univariate analysis in JMP 8.0® for Mac. The T-test/ANOVA was applied for analysis of associations between variables with normal distributions, and the Kruskal-Wilcoxon test was used for non-parametric analysis. All associations between discrete variables were determined using Pearson/Fisher’s test where applicable. The level of significant of association was predetermined at *p* < 0.05 for all analyses.

## Results

### Patient demographics

Of 470 patients approached, a total of 400 agreed to fill out the survey. The mean age of patients surveyed was 38.9 years (SD 14.8 years). Females comprised 52.5% of the survey population; 63.4% of the patients surveyed self-identified themselves as white, 26.3% as black and the rest (10.3%) as belonging to other races. A total of 67% of the respondents disclosed their ethnicity; of these, 71.3% were non-Hispanic non-Latino, 20.5% were Hispanic-Latinos, and 8.2% chose the unknown category. A total of 93.6% respondents were walk-in/ambulatory patients, and the rest were either brought by emergency medical services (EMS) or other means of transport. A total of 53.8% were employed, and 17% of patients did not have any type of insurance. Table 1 shows a detailed distribution of insurance types in the surveyed sample. Of all survey respondents, 79.4% reported having access to a PCP.

**Table 1:** Distribution of insurance types in the survey population

**Variable**	***N *(%)**
No insurance	68 (17.0)
Only private insurance	127 (31.8)
Only Medicare	13 (3.3)
Only Medicaid	89 (22.3)
Private insurance and Medicare	32 (8.0)
Private insurance and Medicaid	22 (5.5)
Medicare and Medicaid	33 (8.3)
All 3 types of insurance	8 (2.0)
Chose not to answer	8 (2.0)

### Reasons for current ED visit

The most common primary complaint in our group of surveyed patients was pain (53.6%). Other complaints included constitutional symptoms such as fever, nausea, cold, headache (14.8%), and minor injuries (14.5%), while 14.1% of patients reported gastrointestinal complaints. Overall 53.6% reported having some kind of pain on presentation, and 22.5% of patients reported that they considered their symptoms acute enough to call 911.

### Access to primary care physician

A total of 315 patients had access to a PCP. A comparison of demographics, employment status and insurance is given in Table 2. Of the patients who had a PCP, 54.8% had called their physicians prior to ED arrival. On analyzing reasons for choosing ED over primary care for the subgroup of patients with PCPs, 44.4% reported coming to the ED as they felt their problems were urgent and required immediate attention, 14.6% reported that they could not get a timely appointment with their PCPs, while 42.9% were actually referred to the ED by their PCP’s office. The most common symptoms for patients with a PCP were GI complaints (*n* = 38, 15.6%). Among respondents who had a PCP, around 64.6% reported that the usual time to available appointment was within 1–3 days, whereas 17.3% reported that their PCP could only give them an appointment for later than 7 days on an average.

**Table 2:** Differences among patient characteristics with regards to access to a PCP

**Variable**	**No access to a PCP (*n *= 82)**	**Access to a PCP (*n *= 315)**	***P *value**
Demographics			
Age (mean, SD)	33.2 (12.8)	40.4 (14.9)	<0.0001
Gender (female)	40 (50.0%)	167 (53.5)	0.57
Race*			
White	36 (46.8)	205 (68.1)	0.001*
Black	30 (38.9)	70 (23.3)	0.005*
Others	11 (14.3)	26 (8.6)	0.14
Ethnicity^#^			
Hispanic	24 (39.3)	30 (14.6)	<0.0001*
NHNL	32 (52.5)	159 (77.6)	<0.0001*
Unknown	5 (8.2)	16 (7.8)	0.92
Employed	46 (56.1)	167 (53.5)	0.68
Some kind of insurance	52 (63.4)	277 (87.9)	<0.0001*

*Seventy-seven subjects with no access to a PCP and 301 subjects with access to PCP disclosed their race; ^#^61 subjects with no access to a PCP and 205 subjects with access to a PCP disclosed their ethnicity.

### Patient’s ED experiences

A total of 385 patients responded to the questions pertaining to their past ED experiences. Of these respondents, 76.4% reported having visited our ED in the past. A total of 17.1% survey responders reported that they had been to other EDs, but this was their first visit to our ED, while 6.5% survey patients said that this was their first ED visit ever. Of the patients who reported having visited our ED in the past, about 20% were “loyal visitors” who had never been to other EDs, while 60.9% were “ED shoppers” who had visited both our and other EDs multiple times.

Among patients with prior ED visits, the self-reported longest waiting time experienced was a mean of 214.3 min (3.6 h) with SD 319.1 min (5.3 h) (median 180 min, IQR 120–270 min). A total of 27.9% of patients reported that they had previously left EDs without being seen (LWBS). Nearly 57.7% of patients said that they would consider leaving without being seen in the future if wait times were too long. The mean acceptable waiting time before leaving the ED without procuring required care was estimated at 221 min (3.7 h) with SD 194 min (3.2 h) (median 180 min, IQR 120–270). The mean age for patients who reported a readiness to leave without being seen was 36.4 years (SD 13.1 years), which was significantly lower than the mean age for patients who did not report the same (mean 42.5 years, SD 16.2 years; *p* = 0.0001). There was no significant association between the readiness to leave without being seen and gender (*p* = 0.22), access to a PCP (*p* = 0.32) and insurance status (*p* = 0.13).

### Triage and waiting room facility preferences

**Figure 1:**
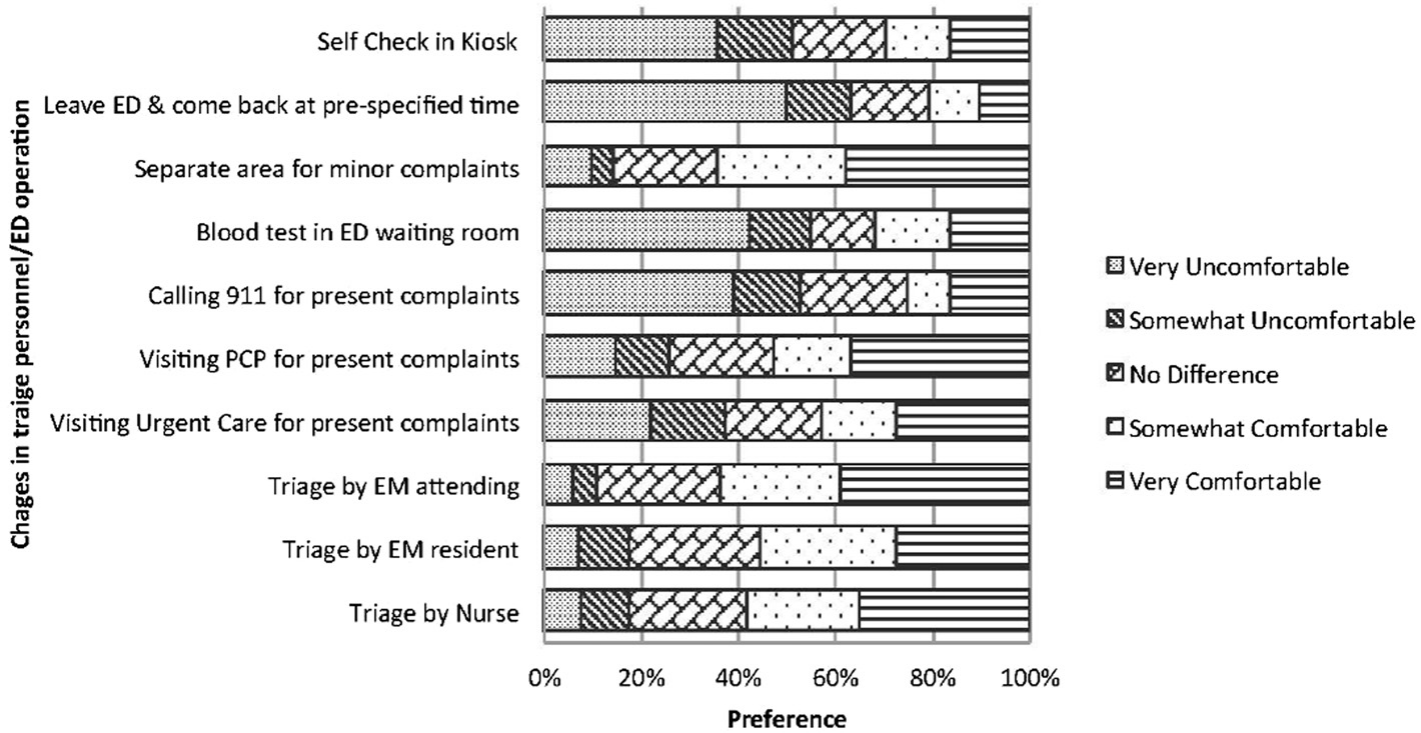
**Patient preferences concerning ED, triage and waiting room facilities.** The comfortability rating of patients concerning different triage personnel in the ED including the attending physician, resident and nurse; visiting the facility for present complaints such as the PCP, urgent care and calling 911, and provision of extra facilities within the ED area such as blood test, separate area for minor complaints, a self check-in kiosk to save time, and leaving the ED and coming back at a pre-specified time.

Survey respondents reported that they were very comfortable being triaged by a physician (39.1%), nurse (35%) and a resident (27.7%) in order of decreasing preference. Figure 1 depicts the above. Figure 1 also displays relative preferences of surveyed subjects for potential operational/structural changes in the ED/waiting room such as initiation of blood tests/draws in the waiting room area, creation of a separate area for managing minor complaints, providing a pre-specified time to return to the ED, a self check-in kiosk, etc.

When asked about their waiting room expectations, nearly 70% of surveyed subjects expressed a need to be given a better estimate of waiting time, and 43.5% wanted better information on reasons for the wait. Further, 30% survey respondents recommended having a coffee and sandwich shop in the waiting area, 16.5% wanted more privacy, 14.8% wanted a quiet area, and 14% expressed the need for better cleanliness.

### Internet use preferences

About 14.1% of patients reported having used the Internet to gather information about their current ailment. Nearly 40.5% of patients reported using the Internet regularly for health-related information. Of the above, 80.8% explored health-related information on the Internet for <2 h/week.

## Discussion

Our observations show that low-acuity patients visiting our tertiary care academic ED are predominantly ambulatory and a significant proportion of them possess medical insurance of some kind. This contradicts the general belief that most patients arriving to the ED with non-urgent complaints are uninsured patients [15]. Our survey also revealed that among these low-acuity ED patients, the proportion of government-insured patients was higher than those of the private-insured or un-insured patients, a finding supported by Zuckerman et al. [16]. The fact that nearly half of the patients in our survey cohort were unemployed may be a reflection of the economic scenario prevalent in the country at the time. Unemployment in Rochester community in 2010 was 8.2% as per the US Department of Labor [17].

Our survey confirmed the presence of a wide range of symptoms within the low-acuity patients waiting in the ED waiting room. However, more than half of these patients reported that they were suffering from some kind of pain. This was similar to a previous study’s results, which showed that only 20% of all ED patients reported a pain score of 0 [18]. In addition to this, nearly 15% of our surveyed population presented with minor injuries, and a similar number complained of constitutional symptoms such as fever and flu-like symptoms. Considering the above, creation of separate fast-track sections of the ED for these illness groups could help to distribute patient load and reduce overall waiting times [19]. This strategy could also potentially improve patient satisfaction, as was evident from our survey wherein subjects revealed a favorable outlook toward the above strategy (38.2% reported being very comfortable with the creation of separate areas in the ED for treating patients with minor complaints).

The survey also displayed an interesting trend in reasons patients gave for preferring EDs over PCP offices. Previous studies have reported that access barriers to primary care are the major determinants for patients to use EDs for minor complaints [20,21]. In our surveyed population, nearly 80% of subjects reported having access to a PCP. Of these, nearly half had contacted their PCP’s office prior to coming to the ED. The proportion of patients calling PCP offices prior to ED arrival has varied in prior studies from as little as 7% to as high as 93% [22-25]. Even for patients with access to a PCP, long waiting times for office appointments lead them to choose EDs over PCP office visits [8,9]. More than 50% of survey respondents in our study reported being comfortable visiting PCPs for their present complaints, and 64% respondents reported being able to get an appointment with their PCP within 3 days. Strikingly though, a third of these patients were referred to the ED by the PCPs or their office personnel themselves. It has been shown that many PCPs or their office personnel routinely refer patients seeking emergent appointments for acute complaints to the ED to maintain their office/clinic schedules [8,26]. Perhaps what we are seeing here is a trend in which PCPs increasingly refer patients to the ED because of increasing practice sizes, decreasing tolerances for uncertainty in diagnoses and increasing insurance capitations on PCP payments [27,28]. It is important however to realize that there could also be other reasons for increased ED referrals. Patients’ complaints over the phone could sound graver than they actually are. Patients’ care may require advanced technology such as an x-ray or ultrasound that may be unavailable at particular times of day at the PCP’s practice. Because our study included patients presenting in the evening, offices may have been closed or closing at the time of the patient’s call. These reasons are legitimate and may be difficult to overcome under the current organization of health-care delivery.

Patient-level factors such as misjudging the severity of illness could also prompt patients to visit ED instead of approaching PCPs. This was evident in our survey results too. A sizeable proportion of the survey population came to the ED because they felt their problems were urgent and required immediate attention. Nearly 44.4% of patients chose the ED over PCP offices because they felt that their symptoms were acute enough to call 911. However, of these only 25% of patients reported having actually called 911. Response to the question about severity of illness could have been post-hoc justification of actions on behalf of the respondents. Cited reasons for severity misperception could also include lack of education, denial, cost, fear, embarrassment, etc [29].

Our survey results also showed that a large proportion of patients repeated their visits to EDs. This may be reflective of the satisfaction offered by the ED. However, there are only four EDs in Rochester area and one additional ED in the suburb of the county. However, only about 20% remained loyal to just one ED. This offers another insight: that patients with non-urgent illnesses keep repeating their ED visits for similar or different non-urgent complaints.

With respect to changes in ED operations, many facilities in this country have introduced varied triage systems such as physician-based triage, treat and release, and employing either ED physicians or ED residents to reduce waiting times in the ED [30,31]. Our survey revealed that patients were most comfortable being seen by ED physicians, triage nurses and ED residents in order of preference. Probably, a system of team triage including physicians, nurses and residents could improve waiting times and increase patient satisfaction [32-34]. Some institutions have also adopted the temporizing strategy of the Casablanca theory, where common blood tests are ordered while patients are waiting in the ED via standing orders to give them a sense of something being done [35]. Patients in our survey population, however, gravitated toward being somewhat uncomfortable with having blood tests done in the waiting area. This may have been due to the perceived lack of privacy or the perception of having tests done sans an expert physician opinion. Our survey population expressed that they would also be uncomfortable if the ED asked them to self-check-in via a kiosk or asked them to return at a later time. This may offer precedent to rethink the move toward the introduction of such strategies to reduce waiting times [36].

It is known that self-reported/perceived waiting times drive actions like 'leaving without being seen’ and decrease overall satisfaction [37]. In our study, although only 28% of respondents had an experience of 'leaving without being seen’ from an ED, an overwhelming 58% reported that they would consider that action if waiting times increased. The longest waiting time that the patients would wait before leaving was estimated to be 3.7 h. The average waiting time before leaving reported in the current literature varies from less than 2 h to over 6 h [38-40]. Younger patients were more likely to express a readiness to leave without being seen if wait times were too long, a finding that has also been shown in the study by Johnson et al. [38]. An increase in the proportion of patients who leave the ED without seeking care also has a direct effect on the reputation of both the hospital and the ED. Gilligan et al. reported that the willingness of patients to revisit the ED decreases with the increase in waiting time [41]. Besides waiting time, the quality of time spent waiting also determines patient satisfaction [42]. In our survey cohort, more than 70% of patients wanted a better estimate of their waiting time, while nearly half of them did not understand why they were waiting. Interventional research has shown that the introduction of pamphlets [43] or videos [44] explaining the working of the ED and the reasons for waiting has a beneficial effect toward patient satisfaction. Some EDs have also started posting expected waiting times on their website or at their triage desk. Improved communication such as described above could increase satisfaction and help increase the tolerance of long ED waiting hours [45,46]. Patients in our survey also expressed the desire for improved privacy, quiet and cleanliness, a snack shop, etc., in the waiting area. In a study in England, even when waiting times were reduced, patients were still dissatisfied with the level of cleanliness in the ED [47]. This brings to view another aspect of the waiting patients: they expect more than just to see the physician.

With the increasing availability of health articles on the Internet, patients increasingly rely on this ever-expanding knowledge network for access to information and expectations of disease treatments. Although acutely ill patients use the Internet less than patients with chronic disease [48], in our study 14% of patients reported using the Internet for their current complaints, while 41% reported using the Internet regularly for health-related information. This presents a largely untapped resource not only for educating patients, but perhaps also for interacting with patients looking to come to the ED with non-acute illnesses.

### Limitations

Our study had some potential limitations. This was a survey of patients who were waiting in the ED to see a physician for more than 15 min post registration. No specific tools like the “Emergency Severity Index” tool (ESI) were used to determine patient’s acuity and resource needs. This could have introduced a measurement bias. However, qualified nurses triaged patients based on accepted criteria for disease acuity. Furthermore, acute patient beds in our ED are separate from low-acuity beds, and crossovers are not allowed. We can therefore reasonably expect that the patients waiting in the ED for more than 15 min most probably did not meet the ESI 1 and ESI 2 criteria and were thus not higher acuity patients. There could have been a recall bias, as this was a self-reported survey, and no attempt was made to recheck the responses from the medical records. The number of interviewers was limited to two for the entire study, and all the information was printed, thus decreasing the variability associated with interviewer bias.

There might have been some sampling error associated with our survey, as some patients (*n* = 70) refused to participate in the study. Although the demographics of these patients were not collected, factors such as increased severity of pain and extremely long waiting times could have influenced patient’s decision to participate in the study. Also, as the survey was administered in English language only and no interpreter was available, this could have led to exclusion of some non-English-speaking patients.

Our study did not attempt to verify whether the PCP directed the patient to the ED for care. More importantly, it was not clear in all cases that the PCP was involved in the decision. Patients could have been directed to the ED by ancillary staff at the PCP’s office. Finally, ours is a tertiary care ED catering to a population of the greater Rochester area with more than 95,000 visits per year, which also offers 24–7 coverage by attending physicians, residents and mid-level providers. Due to the variability in the ED operation and management at other hospital systems, the results of our study should be cautiously generalized to other EDs.

## Conclusions

Our survey revealed that most of the low-acuity patients waiting for care in the ED are more likely to have some form of medical insurance and a significant proportion of them also have a PCP. A significant proportion of patients who had a PCP were referred to the ED by PCP office staff. Our findings also highlight the preferences and expectations of low-severity adult patients waiting for care in the ED, which includes creation of separate areas for minor illnesses and injuries and introduction of physician-based triage. What is perhaps more important is to communicate with these patients to improve their perceptions about the waiting time and the working of the ED.

## Competing interest

The authors declare that they have no competing interests.

## Authors’ contributions

MFK conceived the study idea, participated in the study design, and participated in drafting and revising the manuscript. ARJ conceived the study idea, participated in the study design, collected data, analyzed and interpreted the results, and drafted the manuscript. MJ contributed to the study design, collected data, analyzed and interpreted the results, and drafted and revised the manuscript. SS contributed to critically revising the manuscript. All authors read and approved the final manuscript.
